# Comparative evaluation of posterior percutaneous endoscopy cervical discectomy using a 3.7 mm endoscope and a 6.9 mm endoscope for cervical disc herniation: a retrospective comparative cohort study

**DOI:** 10.1186/s12891-021-03980-9

**Published:** 2021-02-02

**Authors:** Tong Yu, Jiu-Ping Wu, Jun Zhang, Hai-Chi Yu, Qin-Yi Liu

**Affiliations:** Department of orthopaedics, The Second Hospital of Jilin University, Changchun, Jilin Province China

**Keywords:** Cervical intervertebral disc herniation, Minimally invasive spine surgery, Endoscopes, Discectomy, Delta, Keyhole

## Abstract

**Background:**

Posterior percutaneous endoscopy cervical discectomy (p-PECD) is an effective strategy for the treatment of cervical diseases, with a working cannula ranging from 3.7 mm to 6.9 mm in diameter. However, to date, no studies have been performed to compare the clinical outcomes of the use of endoscopes with different diameters in cervical disc herniation (CDH) patients. The purpose of this study was to compare the clinical outcomes of patients with unilateral CDH treated with p-PECD using a 3.7 mm endoscope and a 6.9 mm endoscope.

**Methods:**

From January 2016 to June 2018, a total of 28 consecutive patients with single-level CDH who received p-PECD using either the 3.7 mm or the 6.9 mm endoscope were enrolled. The clinical results, including the surgical duration, hospitalization, visual analog scale (VAS) score and modified MacNab criteria, were evaluated. Cervical fluoroscopy, CT, and MRI were also performed during follow-up.

**Results:**

Tthere was a significant difference in regard to the average identification time of the “V” point (18.608 ± 3.7607 min vs. 11.256 ± 2.7161 min, *p* < 0.001) and the mean removal time of the overlying tissue (16.650 ± 4.1730 min vs. 12.712 ± 3.3079 min, *p* < 0.05) for the use of the 3.7 mm endoscope and the 6.9 mm endoscope, respectively. The postoperative VAS and MacNab scores of the two endoscopes were significantly improved compared with those the preoperative scores (*p* < 0.05).

**Conclusion:**

The application of both the 3.7 mm endoscope and 6.9 mm endoscope represent an effective method for the treatment of CDH in selected patients, and no significant difference can be observed in the clinical outcomes of the endoscopes. The 6.9 mm endoscope shows superiority to the 3.7 mm endoscope in terms of the efficiency of “V” point identification, the removal of overlying soft tissue and the prevention of spinal cord injury. However, the 6.9 mm endoscope may be inferior to the 3.7 mm endoscope in regards to anterior foraminal decompression due to its large diameter; this result needs to be further evaluated with the support of a large number of randomized controlled trials.

## Background

Over a long period, anterior cervical decompression and fusion (ACDF) has seemed to be the gold standard for the treatment of radicular pain triggered by CDH [1]. However, ACDF is also related to various surgical complications, such as dysphonia, dysphagia, recurrent laryngeal nerve palsy, accidental esophageal perforation, hematoma, cerebrospinal fluid leakage, high trauma, slow recovery, implant failure, pseudoarthrosis, bone graft nonfusion, infection, and postoperative adjacent segment degeneration [2–8].

Subsequently, to minimize the surgical complications of ACDF, various surgical techniques have been carried out. Recently, PECD as a treatment for spinal diseases has become favorable. It has the strengths of reducing trauma and accelerating rehabilitation speeds [9–21], and it has a similar short-term clinical benefit to ACDF [22]. PECD can be performed through the anterior approach or posterior approach [21] depending on the site of pathology. Studies have reported that anterior percutaneous endoscope cervical discectomy (a-PECD) possesses the disadvantage of potentially decreasing the intervertebral space postoperatively due to violating the intervertebral disc [23]. However, p-PECD, as a less invasive technique with potential advantages, does not show such shortcomings.

To date, numerous studies have reported p-PECD for the management of cervical disorders [13, 15–21, 23–27] and have studied the inner diameter of the p-PECD working channel, ranging from 3.7 mm to 6.9 mm [17, 22, 28]. However, no studies have been conducted to compare the clinical outcomes of the application of a 3.7 mm endoscope and 6.9 mm endoscope in CDH patients. Therefore, the purpose of this study was to compare the clinical outcomes of patients with unilateral CDH treated by p-PECD with the application of a 3.7 mm endoscope with those of patients treated with a 6.9 mm endoscope.

## Methods

### Patient characteristics

In this retrospective study, we recruited 28 patients with CDH who underwent p-PECD treatment with a 3.7 mm endoscope or 6.9 mm endoscope. All procedures were performed by one surgeon from June 2016 to July 2018. In addition, the demographic characteristics of the 28 patients who were separated into two groups were also collected.

### Inclusion criteria

The indications for p-PECD treatment were as follows: (1) unilateral cervical spondylotic radiculopathy with pain radiating to the upper extremity, (2) MRI and CT scans showing that the foraminal CDH was located lateral to the edge of the spinal cord, from C4–C5 to C7–T1, (3) unilateral symptoms caused by foraminal stenosis, and (4) conservative treatment failure for at least 6 weeks or aggravation of neurological symptoms [21, 29, 30].

### Exclusion criteria

The contraindications for p-PECD treatment were as follows: (1) segmental instability of the cervical spine, (2) multiple-level cervical spinal stenosis, (3) cervical intervertebral discs with calcification, (4) a medial location of the herniated disc, (5) extradural lesions mimicking lateral or foraminal disc herniation, (6) cervical deformity, (7) craniocaudal sequestration of more than half of the vertebral body, (8) anterior osteophytes of the vertebra, (9) bilateral symptoms, and (10) a cause that could not be diagnosed by MRI or CT [23, 29, 30].

### Endoscope instruments

The details are described in Table 1.

**Table 1:** Endoscope Characteristics

Endoscope	OD (mm)	ID (mm)	OA (°)	WL/TL (mm)
6.9 mm endoscope	10 mm	6.9 mm	30	125/205
3.7 mm endoscope	6.9 mm	3.7 mm	15	125/205

*OD* represents outer diameter, *ID* indicates inner diameter, *OV* optics angle, *WL and TL* are the working length and the total length of the endoscope applied, respectively

### Surgical technique

#### 3.7 mm endoscope

**Fig. 1:**
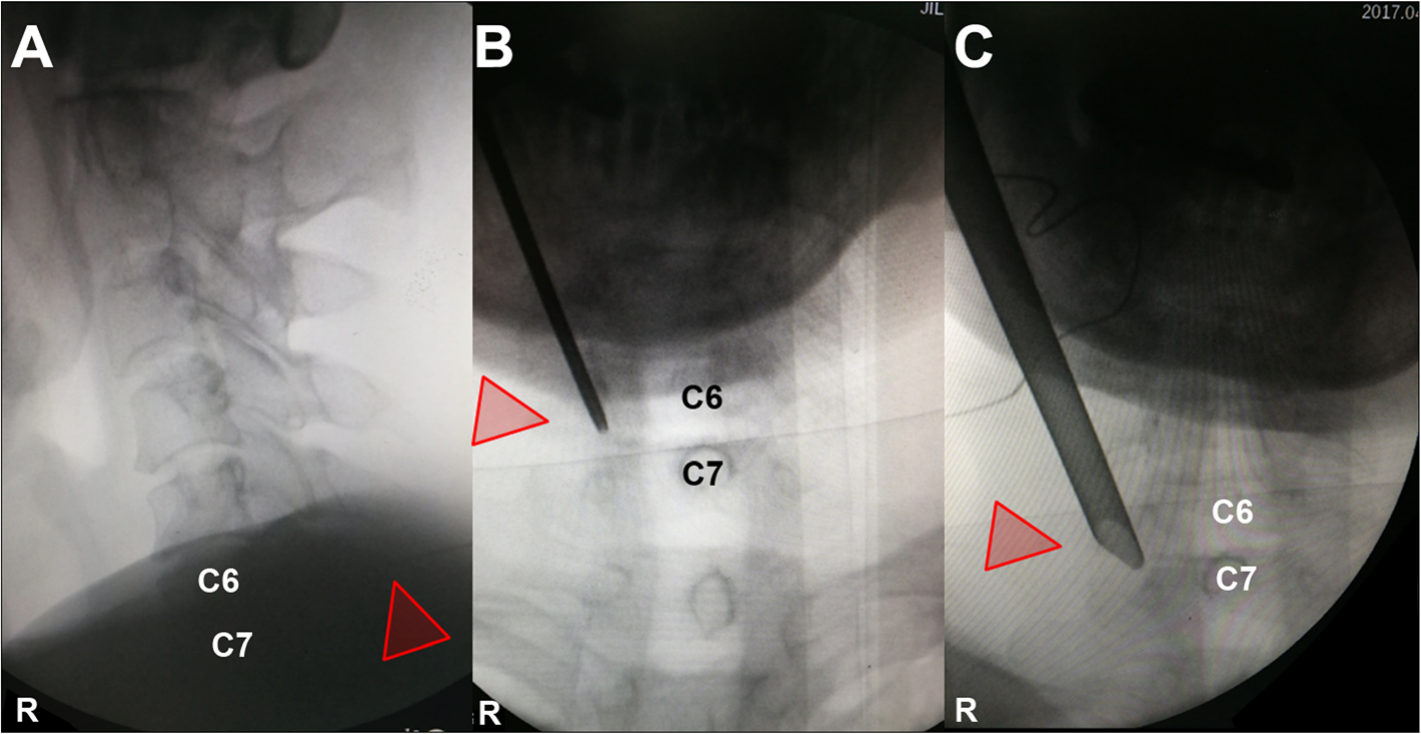
Intraoperative fluoroscopic images during p-PECD with 6.9 mm endoscope. **a** The lamino-facet junction is localized on anteroposterior view; **b** obturator is placed in the “V” point; **c** the working cannula is located over the “V” point

**Fig. 2:**
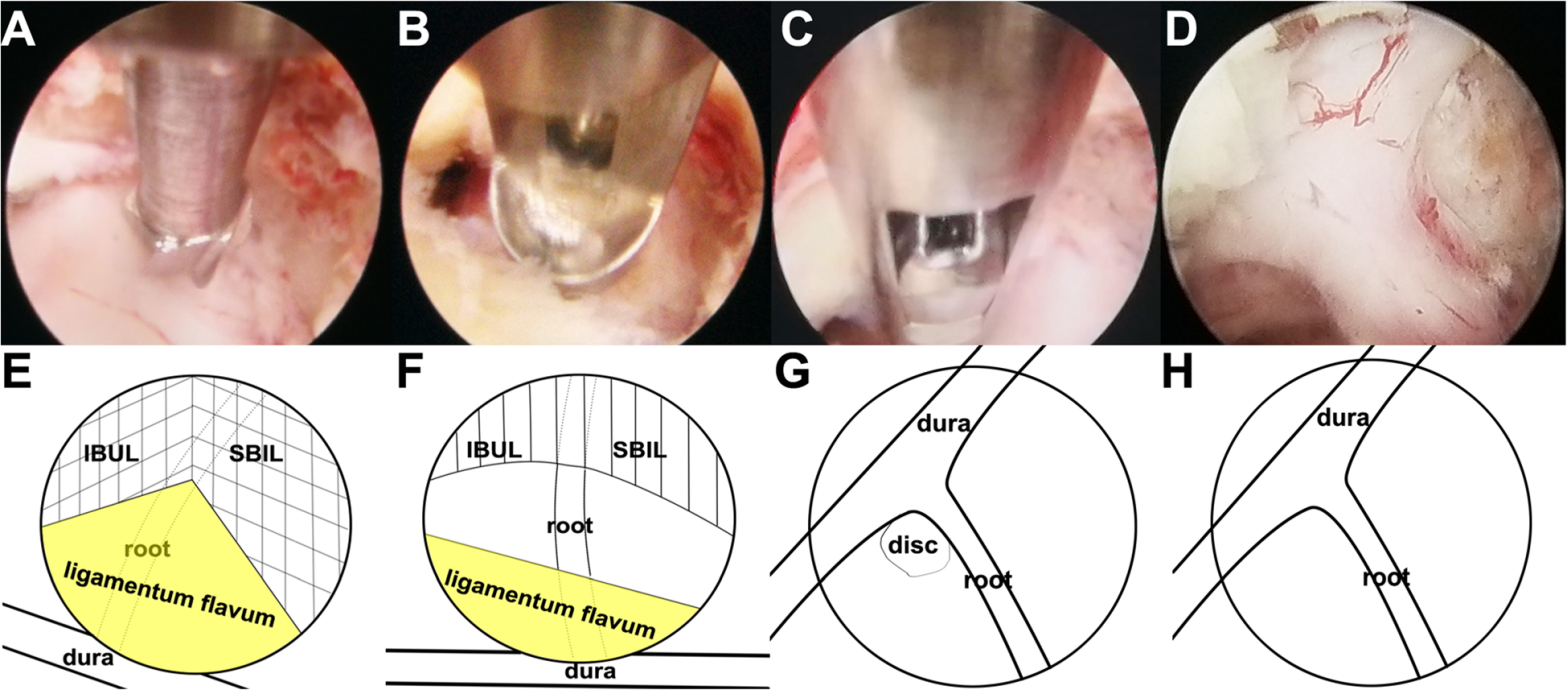
Intraoperative endoscope images during p-PECD with 3.7 mm endoscope. **a** Endoscopic drill was used in performing the laminoforaminotomy; **b** removal of the ligamentum flavum; **c** removing herniated disc through the axilla of the exiting nerve root **c** Intraoperative view after resection of the herniation and free C7 nerve; **e-h** represents (**a-d**), respectively. SBIL indicates the superior border of the inferior laminae; IBUL, inferior border of the upper laminae; LF, ligamentum flavum

**Fig. 3:**
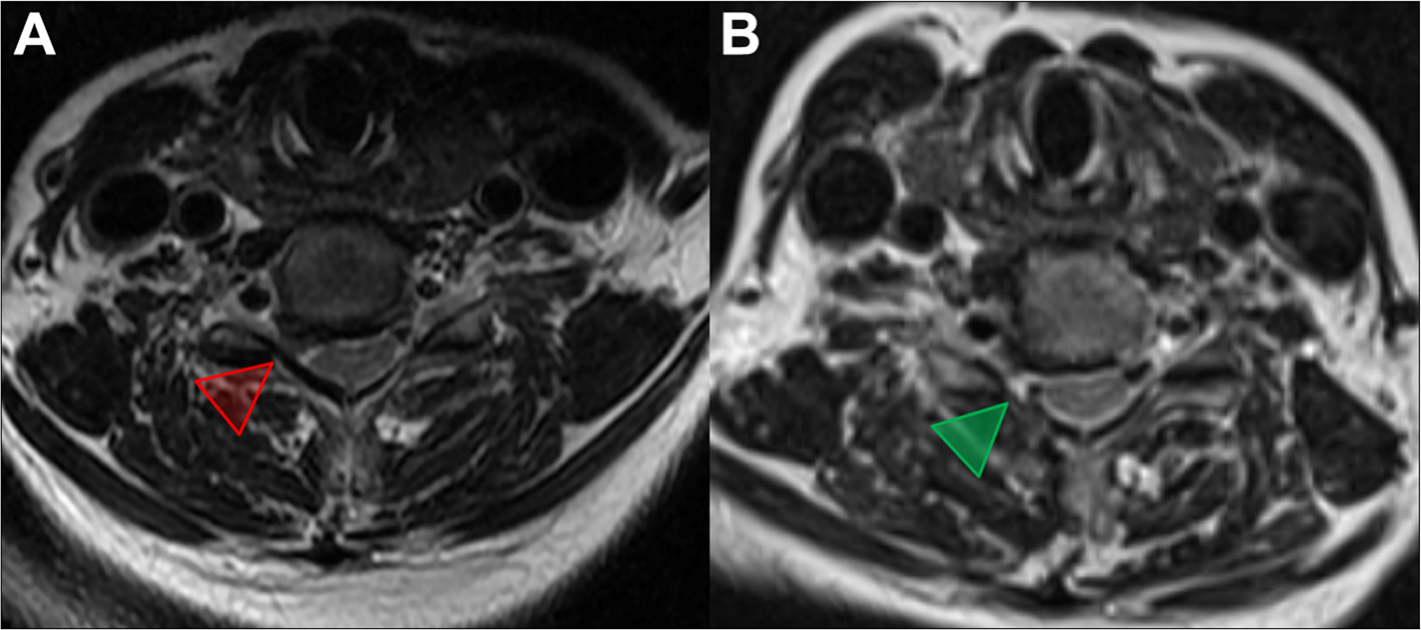
Pre- and postoperative C6-C7 MRI in axial view. **a** Red arrow demonstrates C6-C7 cervical disc herniation; **b** Green arrow indicates that C7 nerve root compression was removed

After general anesthesia, the patient was placed in a prone position. Then, intraoperative neurological monitoring (INM) was performed by a surgeon who specialized in neurosurgery. The surgeon and the assistant stood on the same side as the pathology, and then the endoscope monitor was placed on the opposite side of the pathology. A Kerrison punch or endoscope drill was also applied to conduct laminoforaminotomy or foraminotomy. An endoscope with an inner diameter of 3.7 mm as well as a 30° optics angle was also utilized (Shanghai Maoyu Medical Equipment Co., Ltd., China). Furthermore, all the processes were carried out under continuous irrigation with saline solution. The lamino-facet junction should be observed on the radiograph with a true antero-posterior view to identify the entry point. An 18-gauge Kirschner needle with a length of 25 cm was inserted and placed at the level of the pathology. A 1 cm superficial skin incision was completed. Then, the obturator was implanted and served to palpate the “V” point, which is an anatomical landmark surrounded by the superior border of the inferior laminae, inferior border of the upper laminae, and medial point of the facet joint. Hence, the working cannula was advanced, and then the obturator was removed (Fig. 1). At this point, the endoscope was inserted through the working cannula. A radiofrequency probe (joimax® GmbH, Germany) as well as endoscope forceps were applied to coagulate and remove the overlying soft tissue under continuous irrigation with normal saline. Once the observation of the osseous anatomical structure was completed, the inferior border of the upper laminae was resected with an endoscope drill until the ligamentum flavum was exposed, and then the endoscope drill was directed caudally toward the cervical pedicle and laterally toward the facet joint. Finally, to expose the exiting nerve root, the ligamentum flavum and foraminal ligament were also removed. Subsequently, the underlying disc space was detected with a dissector. Intraoperatively, to prevent excessive removal of the facet joint, a nerve hook was applied to feel the medial wall of the pedicle. After the exiting nerve root was exposed successfully, the intervertebral disc was detected. The herniated cervical disc was removed through the shoulder or axilla of the exiting nerve root according to the lesion location (Fig. 2). It is critical to palpate the exiting nerve root using a nerve hook, and the patient should feel discomfort-free after adequate removal of the intervertebral disc [30, 31]. Figure 3 shows the case of a 60-year-old male with cervical discomfort and radiating pain to the right shoulder and upper extremity. The patient was diagnosed with C6–C7 CDH. The postoperative performance was satisfactory based on the clinical results achieved by the patient.

#### 6.9 mm endoscope

**Fig. 4:**
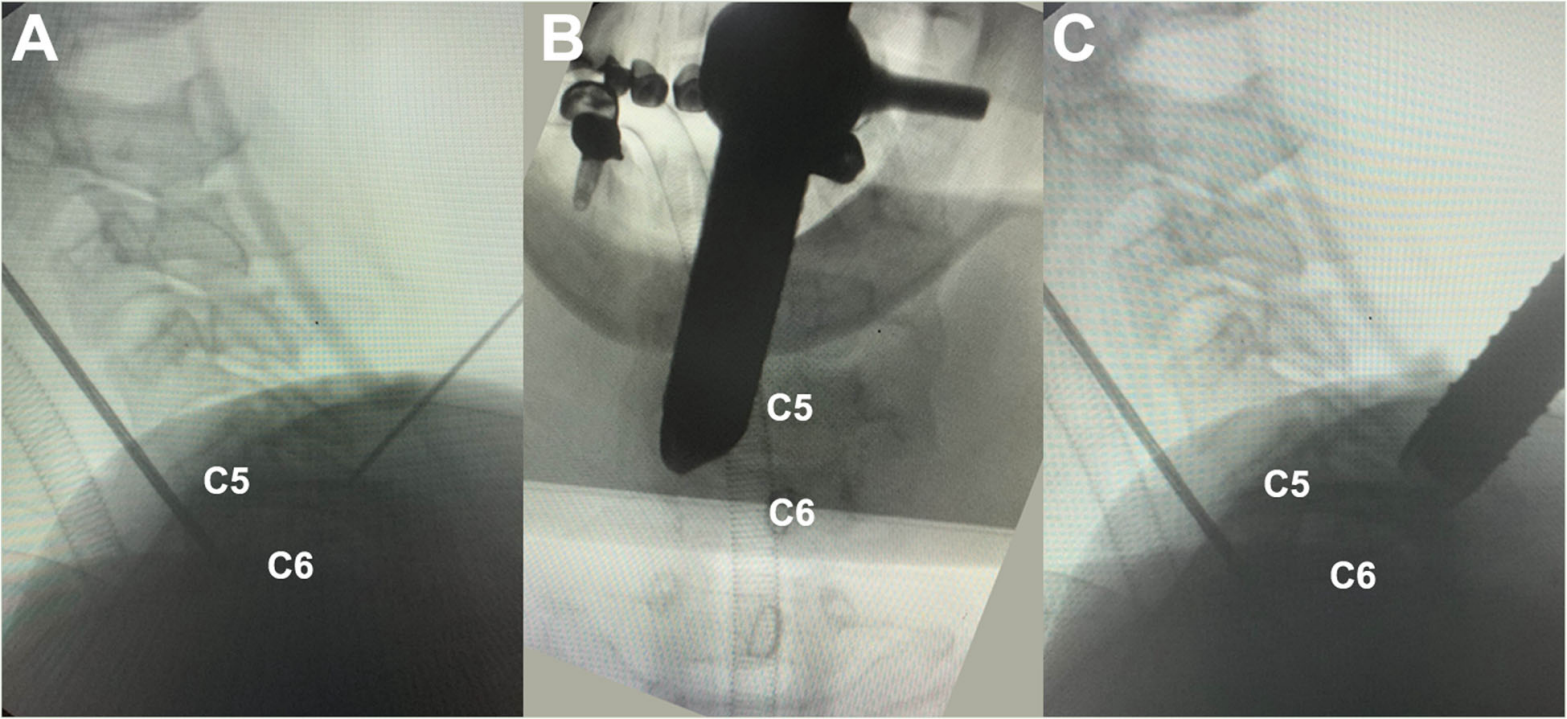
Intraoperative fluoroscopic images during p-PECD with 6.9 mm endoscope. **a** The C5-C6 level was localized on lateral view; **b-c** the working cannula is located over the “V” point on radiographs

**Fig. 5:**
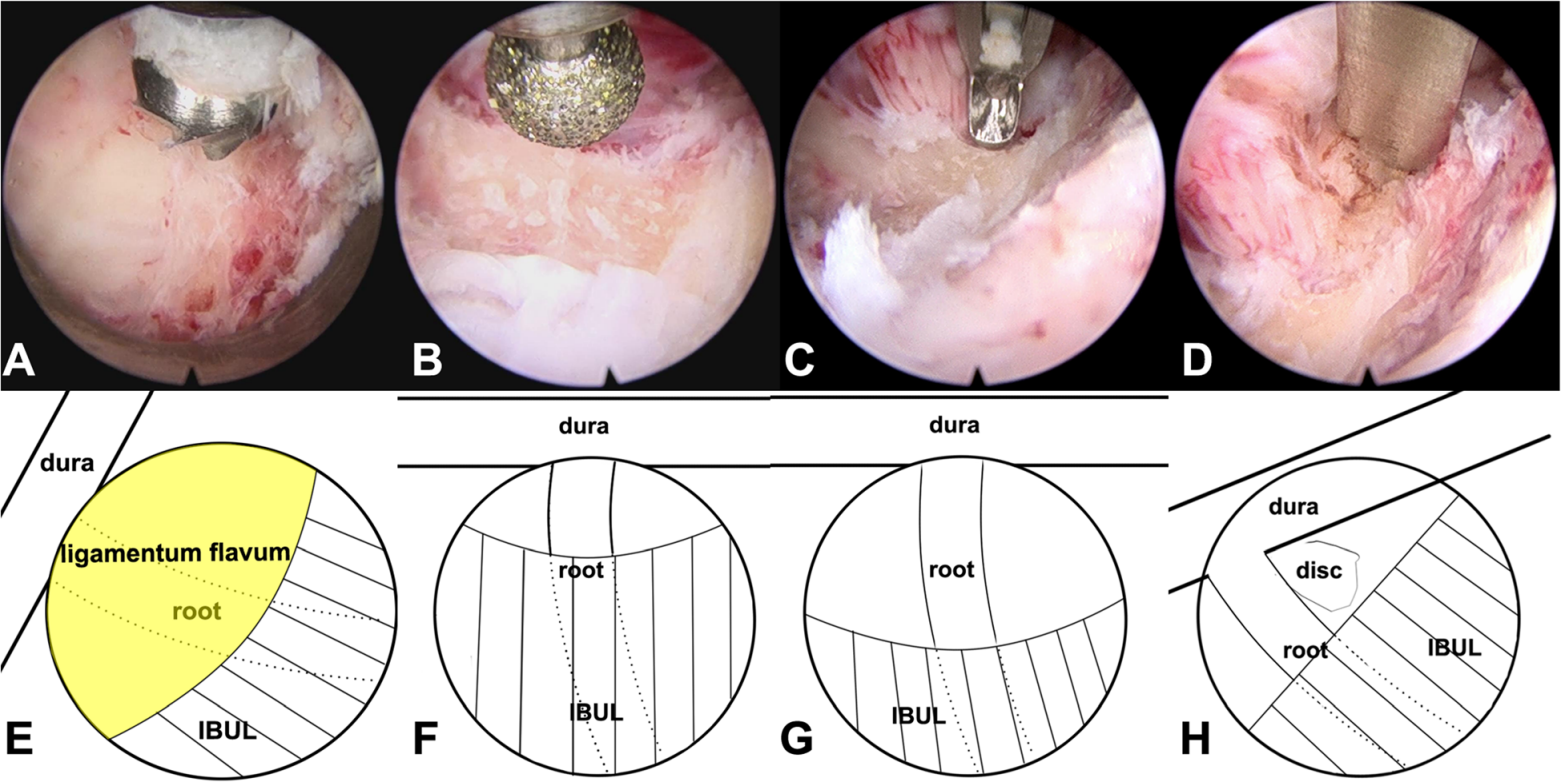
Intraoperative endoscopic images during p-PECD with 6.9 mm endoscope. **a-b** removal IBUL with different endoscopic drill tips; **c** remove IBUL with endoscope forcep; **d** Intraoperative view of dissecting herniated disc through the axilla of the exiting nerve root; **e-h** represents (**a-d**), respectively. IBUL, inferior border of the upper laminae

**Fig. 6:**
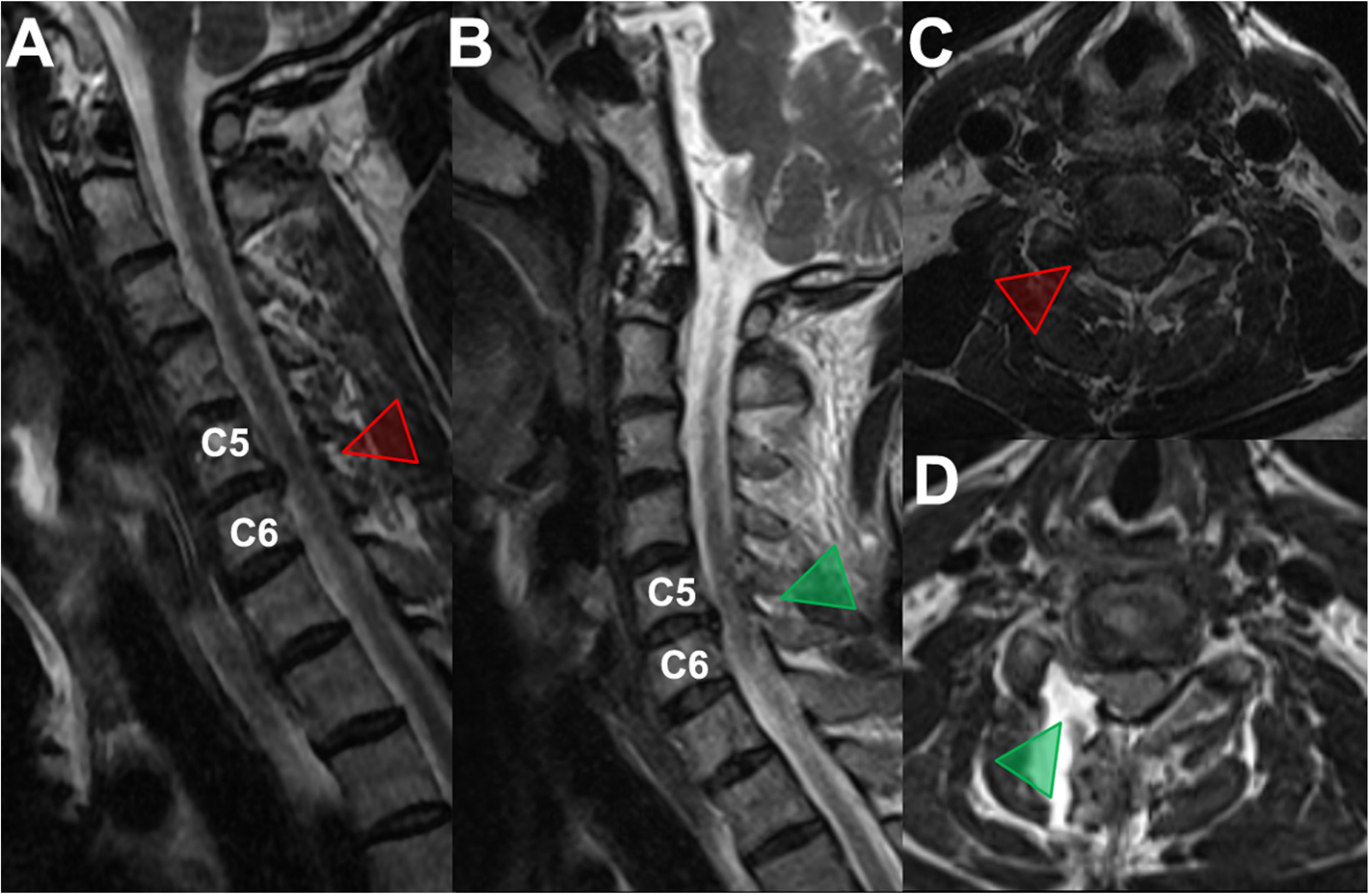
**a**, **c** Pre- and **b**, **d** postoperative MRI images

The surgical tools applied in this technique were slightly different from those applied for the 3.7 mm endoscope. A larger endoscope with a 6.9 mm inner diameter (Fig. 4) was used in this approach (Shanghai Maoyu Medical Equipment Co., Ltd., China). The procedures used for foraminotomy, laminoforaminotomy and discectomy (Fig. 5) remained unchanged compared with those used for the application of the 3.7 mm endoscope. A 54-year-old male patient presented with symptoms such as neck pain and right upper extremity numbness. He was diagnosed with C5–C6 right foraminal CDH (Fig. 6). Good postoperative clinical results were achieved by this patient.

### Statistical analysis

Two-sample t tests, Wilcoxon signed rank tests and Mann-Whitney U tests were applied to compare the parametric data between the 3.7 mm endoscope and the 6.9 mm endoscope. *P* < 0.05 was regarded as the threshold of significance.

## Results

### Patients and surgical characteristics

The demographic characteristics of the 28 patients in the two groups are listed in Table 2.

**Table 2:** Summary of Demographic Data, Clinical Data, and Treatment Level

	3.7 mm endoscope (***n*** = 12)	6.9 mm endoscope (***n*** = 16)	***p*** value*
**Baseline Characteristics**			
Female sex (%)	4 (33)	8 (50)	(*P* > 0.05)
Mean age (range), yr	40.3 (24–79)	40.5 (29–81)	(*P* > 0.05)
Mean duration of symptoms (range), wk	14 (7–48)	16 (3–39)	(*P* > 0.05)
**Indications for surgery**			
Radiculopathy	12	16	(*P* > 0.05)
**Treatment level**			
C4–C5 (%)	2 (16)	1 (6)	(*P* > 0.05)
C5–C6 (%)	3 (24)	4 (25)	(*P* > 0.05)
C6–C7 (%)	7 (60)	10 (63)	(*P* > 0.05)
C7–T1 (%)	0 (0)	1 (6)	(*P* > 0.05)

*p value represents the significant difference in the basic characteristics between patients with the use of the 3.7 mm endoscope and patients with the use of the 6.9 mm endoscope, and *P* > 0.05 indicates that no statistically significant differences were observed

**Fig. 7:**
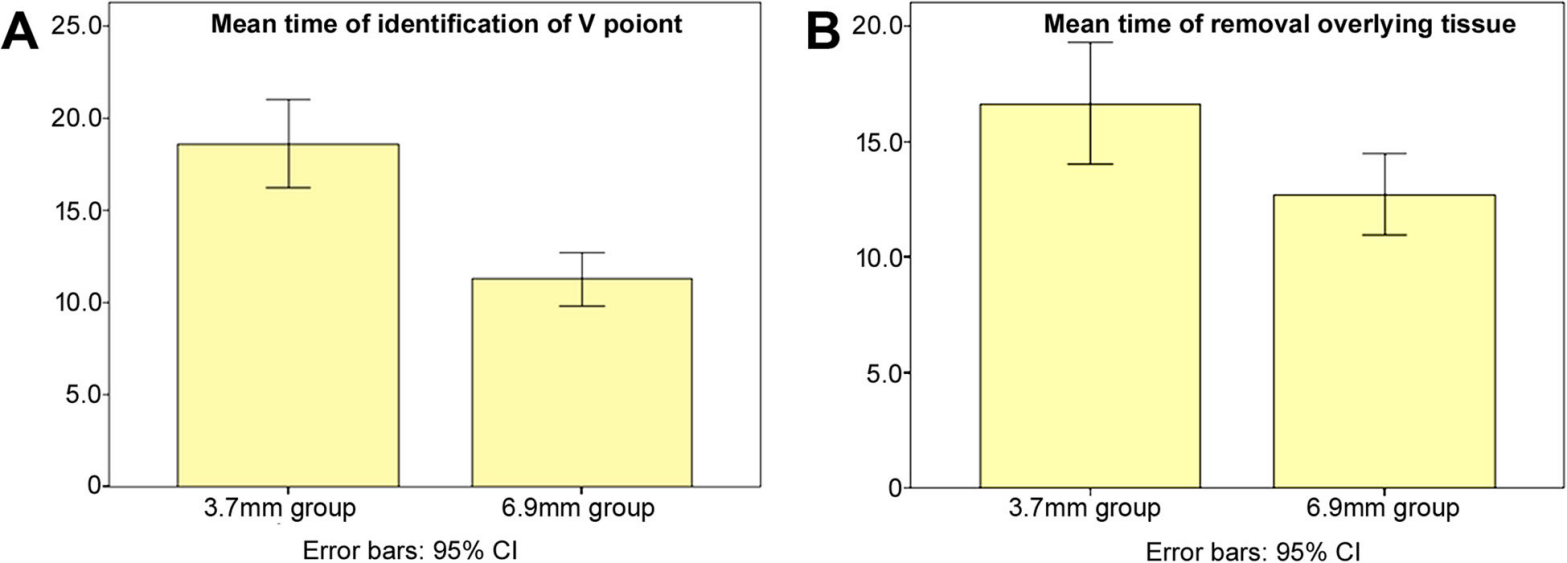
**a** The mean time of “V” point identification in the use of 3.7 mm endoscope and 6.9 mm endoscope was compared; **b** The mean time of the removed overlying tissue in the use of 3.7 mm endoscope and 6.9 mm endoscope was also compared

The surgical characteristics and complications are shown in Table 3. The blood loss of both groups was negligible. The mean hospital stay did not show a significant difference between patients with the use of the 3.7 mm endoscope and patients with the use of the 6.9 mm endoscope. However, the mean surgical duration for patients with the 3.7 mm endoscope was 76.5 min, while it was 61.5 min for patients with the 6.9 mm endoscope (*P* < 0.05). Moreover, there was also a significant difference in regard to the average identification time of the “V” point (18.608 ± 3.7607 min vs. 11.256 ± 2.7161 min, *p* < 0.001) and the mean removal time of overlying tissue (16.650 ± 4.1730 min vs. 12.712 ± 3.3079 min, *p* < 0.05) (Fig. 7) between patients with the 3.7 mm endoscope and patients with the 6.9 mm endoscope.

**Table 3:** Operative Characteristics and Postoperative Course

	3.7 mm endoscope (***n*** = 12)	6.9 mm endoscope (***n*** = 16)	***p*** value*
**Surgical Characteristics**			
Mean surgical duration	76.5 (58–131)	61.5 (40–98)	(*p* < 0.05)
Mean hospital stay (range)	5.1 (2–8)	4.8 (3–6)	(*P* > 0.05)
**Total complications**			
Dura injury	0	1	(*P* > 0.05)
Postoperative headache	0	0	–
Repeated surgery	0	0	–
Postoperative hematoma	0	0	–
Neurological deterioration	0	0	–

*P value represents the significant difference in the surgical characteristics among patients with the application of the 3.7 mm and 6.9 mm endoscopes, and *P* < 0.05 was regarded as the threshold of significance

### Complications

One case in this study described a patient who suffered from nerve root outer membrane injury from the 6.9 mm endoscope (1 of 16, 6.25%). However, no cerebrospinal fluid leakage during the operation or neurological deterioration was detected postoperatively. There were also no other severe surgical complications reported in either group, such as carotid artery injury, recurrent laryngeal nerve injury, esophageal injury or infection. None of the 28 patients experienced recurrence in the follow-up.

### Clinical outcomes

**Fig. 8:**
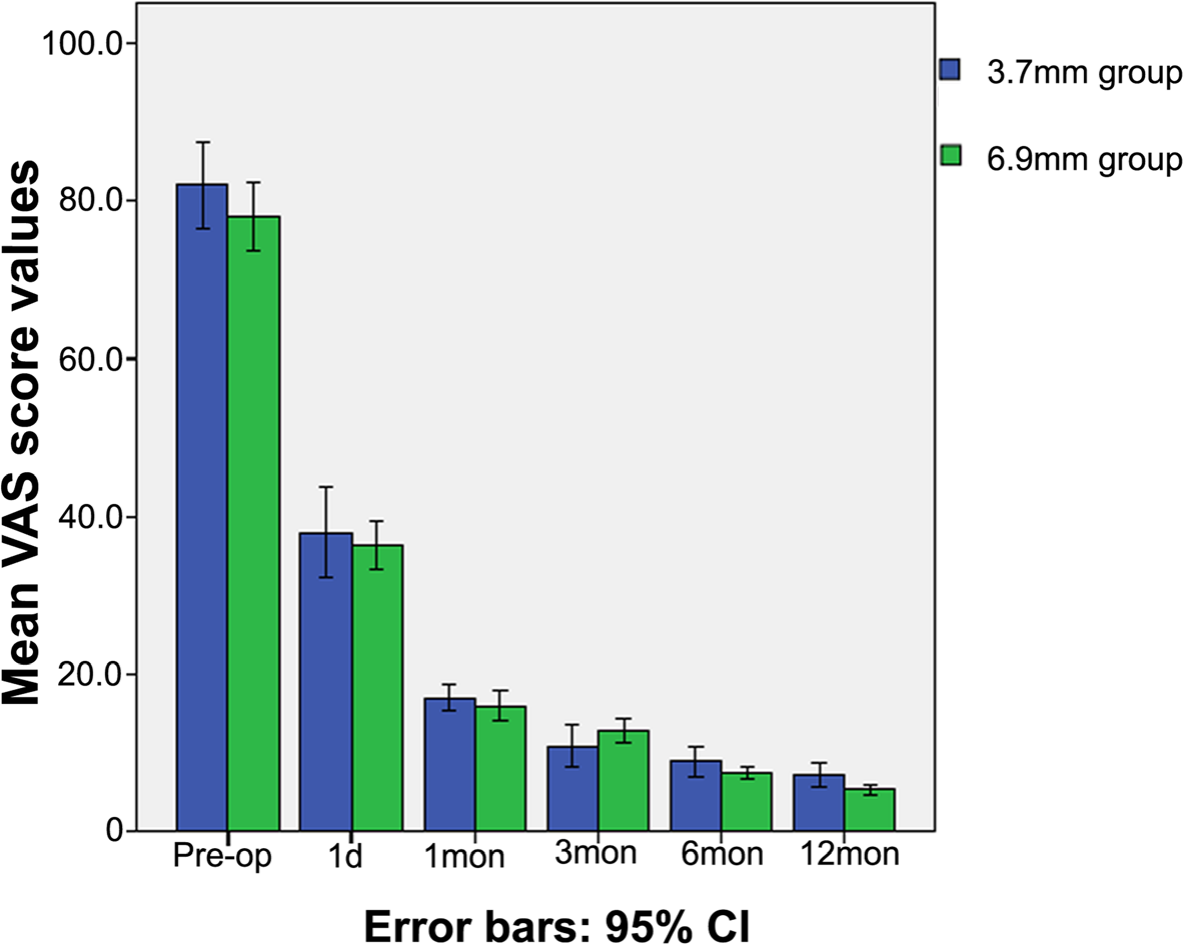
The course of the arm and neck pain in both groups, which was rated using the mean visual analogue scale values

**Fig. 9:**
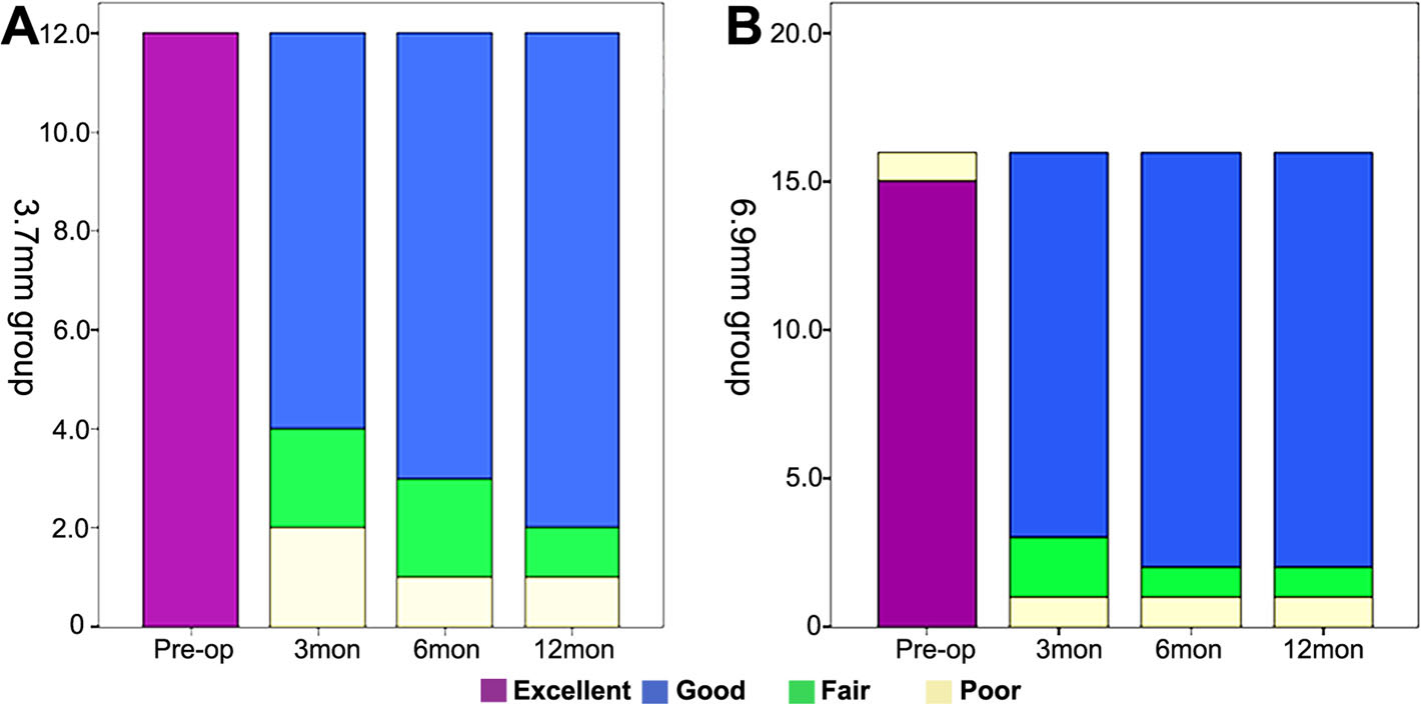
The clinical results of the **a** cervical discectomy with the use of 3.7 mm endoscope and **b** cervical discectomy with the use of 6.9 mm endoscope according to the modified MacNab criteria

All 28 patients completed the follow-up visits. Two out of the 28 patients, with one patient each in the two groups, showed no significant pain relief at the 12-month follow-up. The VAS scores (Fig. 8) and the modified MacNab criteria (Fig. 9) of the 28 patients were evaluated both preoperatively and postoperatively. No significant differences in the mean VAS score or outcomes evaluated were detected using the modified MacNab criteria between the 3.7 mm endoscope and 6.9 mm endoscope. In addition, no difference was found in complications between the two groups (*P* > 0.05).

### Follow-up

For all patients, the follow-up was always performed 1 day postoperatively and 1, 3, 6 and 12 months postoperatively. The VAS scores and the modified MacNab criteria were calculated preoperatively and postoperatively for the purpose of evaluating the clinical outcomes. Cervical CT or MRI was performed on all the patients during the follow-up period.

## Discussion

In 1944, Spurling et al. first described the effectiveness of p-PECD for the treatment of cervical foraminal stenosis induced by lateral CDH or osteophytes [32]. Studies have proven that p-PECD is an effective treatment for cervical diseases, and the inner diameter of the working cannula has ranged from 3.7 mm to 6.9 mm [17, 22, 28]. In our opinion, different diameters of the working cannula may lead to different surgical efficiencies. However, no comparative studies have been conducted to analyze the clinical outcomes of the application of a 3.7 mm endoscope or a 6.9 mm endoscope for p-PECD in patients with CDH. In this study, we analyzed the clinical results of 28 consecutive patients who were diagnosed with unilateral CDH and underwent p-PECD using a 3.7 mm endoscope or 6.9 mm endoscope.

### Anesthesia

Studies have suggested that both local and general anesthesia are effective strategies for PECD [17, 22, 23, 33]. Wan et al. [17] announced that the use of local anesthesia in selected patients with CDH is a promising and feasible alternative. However, local anesthesia still has some unavoidable shortcomings, such as discomfort and psychentonia during the operation. Moreover, if the patient is awake, the noise produced by the surgical instrument may result in an elevated blood pressure, an increased heart rate, or an unpleasant surgical experience [17]. General anesthesia has been explored in several previous studies, which all confirmed that it could offer patients a comfortable experience during p-PECD surgery [22, 23, 33].

In the present cohort, to minimize intraoperative anxiety and pain as well as to attain better cooperation by patients, general anesthesia was carried out in all patients. In addition, INM technology was applied in this study to prevent iatrogenic neurological deterioration intraoperatively. The detailed method has been described by Yu et al. [17, 34]. No nerve compromise was observed in either of the groups postoperatively, and we attribute these positive results to the reasonable choice of anesthesia method and the application of INM.

### Clinical results

The mean hospital stay for traditional posterior foraminotomy or ACDF in China is usually more than 7 days [23]. In our study, the mean hospitalization times of patients in whom the 6.9 mm endoscope or 3.7 mm endoscope was used were 5.1 (from 2 to 8) and 4.8 (from 3 to 6) days, respectively, and both groups showed an improvement in hospital stay compared with the average results in China. Since all the surgeries in this study were performed under general anesthesia, it took approximately 2 days to complete the preoperative examination and the assessment of the general condition of the patient to meet the requirements for conducting general anesthesia. Under normal circumstances, patients were discharged 2 days after the completion of the postoperative observation. Thus, the total length of hospital stay was approximately 5 days. However, no significant difference was observed between patients with the 3.7 mm endoscope and patients with the 6.9 mm endoscope in terms of the average hospital stay period (*P* > 0.05). However, the use of the 3.7 mm endoscope (76.5 min) required longer operative times than the use of the 6.9 mm endoscope (61.5 min). We believe that this result may be because the small-diameter working cannula can only accommodate smaller-diameter endoscope instruments, such as RF probes, forceps and drills, which obviously limits the efficient identification of the “V” point, the removal of overlying soft tissue and the procedure of laminoforaminotomy.

On the basis of previous surgical experience [23, 35, 36], the average VAS score after surgery was significantly lower with the application of both endoscopes; however, the difference in the average VAS scores between the use of the 3.7 mm endoscope and the 6.9 mm endoscope was not obvious (*P* > 0.05). Moreover, taking the modified MacNab criteria into consideration, the proportion of a satisfied result (excellent or good recovery) improved during the follow-up visit in the application of both endoscopes; nevertheless, the difference between the 3.7 mm endoscope and 6.9 mm endoscope was not significant (*P* > 0.05). Therefore, the clinical outcomes of both endoscopes suggest similar efficiencies.

### Operation technique

#### Identification of the “V” point

The identification of the V-point is an extremely critical operation step in determining the success or failure of p-PECD. Furthermore, the accurate and rapid confirmation of the V-point can provide sufficient confidence for surgeons in proceeding to the next step. In our study, the identification of the V-point was easier with the application of the 6.9 mm endoscope than with the application of the 3.7 mm endoscope (18.608 ± 3.7607 min vs. 11.256 ± 2.7161 min, *p* < 0.001), which may be attributed to the large diameter of the working cannula in the 6.9 mm endoscope.

#### Potential of spinal cord injury

In this study, the application of neither the 3.7 mm nor the 6.9 mm endoscope resulted in the surgical complication of spinal cord damage. However, our corresponding author argues that the use of the 3.7 mm endoscope has a higher risk of spinal cord injury than the use of the 6.9 mm endoscope. The minimal working cannula of the 3.7 mm endoscope has the potential to become trapped in the spinal canal via the iatrogenic hole, thus damaging the spinal cord. Moreover, the 6.9 mm endoscope has a working cannula with a wider outer surface, which can prevent it from being negligently inserted into the spinal canal, eventually increasing the safety of the operation. This idea was also agreed upon by Lin et al. [20], who suggested that increasing the outer diameter of the working cannula could reduce the risk of spinal cord injury.

#### Anterior decompression

The application of the 3.7 mm endoscope is better than that of the 6.9 mm endoscope in terms of anterior decompression of the intervertebral foramen due to the smaller outer diameter of the working cannula, which functions to reduce compression of the spinal cord. In contrast, the delta working channel, which has a large inner diameter, may lead to spinal cord injury.

### Complications

Surgical-related complications, including headache, neck pain, dural damage, nerve root or spinal cord injury, seizures or neurological deterioration due to the highly increased cervical epidural pressure resulting from continuous saline irrigation, intraoperative bleeding or postoperative epidural bleeding, instability caused by surgery and infections, can occur after p-PECD for CDH patients [13, 23].

In 2007, Ruetten et al. [22, 30] stated a complication rate of 3% in 89 patients who underwent p-PECD, and in 2008, he reported three postoperative complications associated with transient, dermatome-related hypesthesia. In 2009, Joh et al. [37] demonstrated in a prospective study that 8 of 28 patients complained of neck pain caused by the increased pressure of the continuous irrigation system. In 2014, Yang et al. [23] observed a patient with transient pain on the contralateral side, which was due to excessive myelin dissection, and concluded that an incidence of 4.8% (2/42) of such symptoms occurred in patients who underwent p-PECD. In 2018, Wu et al. [27] reported that two patients suffered from bluntness of the pupillary light reflex, loss of consciousness, muscle weakness in the extremities and weak spontaneous respiration among those who underwent p-PECD under local anesthesia. The C6 lamina was perforated with the spinal needle, which then led to anesthetics passing through the iatrogenic hole and entering the subarachnoid space.

In the present cohort, the nerve root outer membrane was torn in one patient in whom the 6.9 mm endoscope was applied, but no cerebrospinal fluid leakage was observed during the operation, and no neurological deterioration was observed postoperatively. No other surgical complications were observed in either of the groups. The overall incidence of surgical complications in our study was 3.7% (1/28), and this result is similar to the results of previous studies [22, 23].

### Limitations

Despite all the positive clinical outcomes achieved in this study, there were still many limitations. The limitations of our study include the following: the small sample size, the lack of randomization, the use of a single surgeon, the deficiency of multicenter research and the comparably short-term follow-up period. In summary, multicenter randomized controlled trials with large sample sizes and long-term follow-up visits should be further established.

## Conclusion

In the present study, there were no significant differences in the clinical outcomes between the use of the two endoscopes. The applications of the 3.7 mm endoscope and 6.9 mm endoscope have their respective advantages. In terms of determining the “V” point, the removal of overlying soft tissue as well as the prevention of spinal cord injury using the 6.9 mm endoscope may be preferable. However, using the 3.7 mm endoscope may be a better option for anterior decompression of the intervertebral foramen. Overall, p-PECD, including the application of both the 3.7 mm endoscope and 6.9 mm endoscope, is a reliable alternative management strategy for CDH.

## Data Availability

The datasets used during the current study are available from the corresponding author on reasonable request.

## Abbreviations

CDH: Cervical disc herniation
p-PECD: Posterior percutaneous endoscope cervical discectomy
VAS: Visual analogue scale
ACDF: Anterior cervical decompression and fusion
a-PECD: Anterior percutaneous endoscope cervical discectomy
INM: Intraoperative neurological monitoring
